# Serum homocysteine is associated with tubular interstitial lesions at the early stage of IgA nephropathy

**DOI:** 10.1186/s12882-021-02632-3

**Published:** 2022-02-23

**Authors:** Zizhen Li, Qianqian Han, Hongbo Ye, Jiajia Li, Xiaona Wei, Rui Zhang, Qiuyan Huang, Yanchun Xu, Guanxian Liu, Bin Li, Qiongqiong Yang

**Affiliations:** 1 Department of Nephrology, Sun Yat-sen Memorial Hospital, Sun Yat-sen University, No.107 Yanjiang West Road, Yuexiu District, Guangzhou, Guangdong People’s Republic of China; 2 Clinical Trials Unit, The First Affiliated Hospital, Sun Yat-sen University, No.58 Zhongshan 2nd Road, Yuexiu District, Guangzhou, Guangdong People’s Republic of China

**Keywords:** IgA nephropathy, Serum homocysteine, Tubular interstitial lesions

## Abstract

**Background:**

The association between homocysteine (Hcy) and IgA nephropathy (IgAN) is not well understood. We aimed to investigate the relationship between Hcy and clinicopathologic features in IgAN patients.

**Methods:**

A total of 337 IgAN patients and 150 sex- and age- matched healthy controls were enrolled in this single-center retrospective study. According to Hcy ≤ 10 μmol/L or > 10 μmol/L, patients were divided into low and high Hcy groups. Multivariate logistic regression was performed to explore the risk factors for elevated Hcy.

**Results:**

Serum Hcy was higher in IgAN patients than in healthy controls [11.6 (9.1,15.3) vs. 8.8 (7.5,10.6) μmol/L, *P* < 0.001], unanimously in the subgroup of 156 patients with a normal estimated glomerular filtration rate (eGFR) (≥ 90 ml/min/1.73 m^2^) [9.9 (7.6,12.4) vs. 8.8 (7.5,10.6) μmol/L, *P* < 0.001]. Compared to the low Hcy group, serum creatinine (Scr), blood urine nitrogen (BUN), uric acid (UA), endocapillary hypercellularity (E) and tubular atrophy/interstitial fibrosis lesion (T) were higher in the high Hcy group. Hcy levels were positively correlated with Scr, BUN, UA, 24-h urine protein, and E and T lesions, but negatively correlated with eGFR and superoxide dismutase (SOD). In the subgroup with normal eGFR, patients with higher Hcy were persistent with higher Scr, BUN and T lesions. A multivariate logistic regression model showed that the risk of elevated Hcy in patients with pathological T increased by 2.87-fold. T lesions could better predict high Hcy, with an odds ratio (OR) of 14.20 in the subgroup with normal eGFR.

**Conclusions:**

Pathologic T was an independent risk factor associated with elevated Hcy, especially at the early stage of IgAN.

**Supplementary Information:**

The online version contains supplementary material available at 10.1186/s12882-021-02632-3.

## Background

Homocysteine (Hcy) is a sulfhydryl amino acid situated at a branch point of methionine metabolism that is metabolized via two interrelated processes of remethylation and transsulfuration [1]. Numerous studies have found that elevated serum Hcy is a nontraditional independent risk factor for cardiovascular disease (CVD) [2, 3]. Elevated serum Hcy is a common finding in patients with chronic kidney disease (CKD) [4], which is attributed to reduced renal clearance of circulating Hcy and impaired Hcy metabolism [5]. Furthermore, elevated serum Hcy was confirmed to be related to the prevalence and progression of CKD [6, 7].

IgA nephropathy (IgAN) is the most common primary glomerulonephritis [8]. Approximately 30–40% of IgAN patients eventually develop end-stage renal disease (ESRD) within 20–30 years of renal biopsy [9]. A small retrospective study from Duan et al. revealed that elevated Hcy was associated with poor renal outcome in IgAN patients [10]. Another study by Mendelian randomization (MR) analysis observed positive effects of Hcy on serum creatinine, blood pressure (BP), and pathogenic T lesions in IgAN patients [11]. However, only the two small-sample studies mentioned above have investigated the relationship between Hcy and IgAN patients. Clinical research on the relationship between Hcy and pathology has not yet been highlighted.

In this study, we investigated the relationship between Hcy and clinicopathologic characteristics in IgAN patients in our center, especially at an early stage with a normal estimated glomerular filtration rate (eGFR) (≥ 90 ml/min/1.73 m^2^).

## Methods

### Study design and participants

This was a single-center retrospective study. Patients (age ≥ 14 years) with biopsy-based diagnosis of primary IgAN between April 2014 and March 2021 in the Sun Yat-sen Memorial Hospital of Sun Yat-sen University were enrolled. Patients with secondary IgAN, such as systemic lupus erythematosus, Henoch-Schönlein purpura, anti-neutrophil cytoplasm antibody (ANCA)-associated vasculitis, diabetic nephropathy or hepatic diseases were excluded from the study. Patients with fewer than 8 glomeruli in biopsy samples, incomplete data and CVDs such as stroke, coronary atherosclerotic heart disease, aortic aneurysm, and aortic dissection, were also excluded. Patients with Hcy ≤10 μmol/L and Hcy > 10 μmol/L were classified into the low Hcy group and high Hcy group, respectively [11]. A total of 150 sex- and age- matched healthy controls were recruited from the Center of Health Examination in order to explore whether serum Hcy was elevated in IgAN patients. This study was approved by the Medical Ethics Committee of Sun Yat-sen Memorial Hospital, Sun Yat-Sen University (approval number: No. SYSEC-KY-KS-2020-187), and exemption from informed consent of all patients was agreed upon.

### Demographic and clinicopathologic data collection

Demographics and clinicopathologic data were retrieved from the medical record system. All laboratory indicators, including serum Hcy, were collected and measured at the time of biopsy and retrospectively analyzed. Demographic data included sex, age, systolic blood pressure (SBP), diastolic blood pressure (DBP), mean arterial pressure (MAP) and history of hypertension (HBP). Laboratory indicators included 24-h urine protein, Hcy, serum creatinine (Scr), blood urine nitrogen (BUN), uric acid (UA), hemoglobin (Hb), serum albumin (ALB), fasting glucose (Glu), cholesterol (CHOL), triglyceride (TG), low-density lipoprotein-cholesterol (LDL), high-density lipoprotein-cholesterol (HDL), superoxide dismutase (SOD), high sensitivity C reaction protein (hsCRP), erythrocyte sedimentation rate (ESR), immunoglobulin A (IgA), immunoglobulin M (IgM), immunoglobulin G (IgG), complement 3 (C3), and complement 4 (C4). MAP was calculated as DBP plus a third of the pulse pressure. HBP was defined as SBP ≥ 140 mmHg and/or DBP ≥ 90 mmHg or requirement for antihypertensive therapy. The eGFR was calculated using the Chronic Kidney Disease Epidemiology Collaboration (CKD-EPI) equation [12]. Blood samples were routinely collected in the morning after overnight fasting when hospitalized, and serum Hcy levels were measured using a Backman AU5831 automatic biochemistry analyzer (USA) before biopsy.

Renal biopsy specimens were examined by light, immunofluorescence, and electron microscopy. Two pathologists independently evaluated histopathological manifestations according to the Oxford classification system of IgAN [13]. Mesangial hypercellularity (M), endocapillary hypercellularity (E), segmental glomerulosclerosis (S), tubular atrophy and interstitial fibrosis (T) and crescents (C) were recorded in detail.

### Statistical analysis

Continuous variables with a normal distribution are expressed as the mean ± standard deviation and were compared via Student’s t test. Skewed distributed continuous variables were expressed as medians (interquartile ranges) and compared with the Mann–Whitney U test or the Kruskal–Wallis test. Categorical variables were expressed as frequencies (percentages) and compared via the chi-squared test. The *P* value for multiple comparisons was corrected according to the Bonferroni method. Spearman’s rank correlation was applied to detect the association between Hcy and clinicopathological features in IgAN patients. Univariate and multivariate logistic regression analyses were performed to identify the risk factors for elevated Hcy. A multivariate logistic regression model was created using stepwise forward LR to identify the variables that were independently associated with elevated Hcy selected from the variables with *P* < 0.1 in univariate logistic regression analysis. We further carried out analysis in the subgroup with normal renal function patients based on eGFR ≥90 ml/min/1.73 m^2^ to modify the effect of renal impairment. The data were analyzed using the SPSS 25.0 package (Chicago, IL, USA). All the *P* values were two-tailed, and *P* < 0.05 was considered significant in all statistical tests.

## Results

### Serum Hcy and clinicopathologic parameters of IgAN patients

A total of 337 IgAN patients aged 34.0 (27.5, 44.0) years entered the study, and 40.1% (*n* = 135) were male. The age of the healthy controls was 36.0 (30.0, 40.0) years, and 42.7% (*n* = 64) were male. The median levels of eGFR and MAP were 88.1 (62.8, 103.4) ml/min/1.73 m^2^ and 94.7 (88.7, 106.3) mmHg, respectively. The median level of 24-h urine protein was 0.6 (0.2, 1.7) g/24 h.

**Fig. 1:**
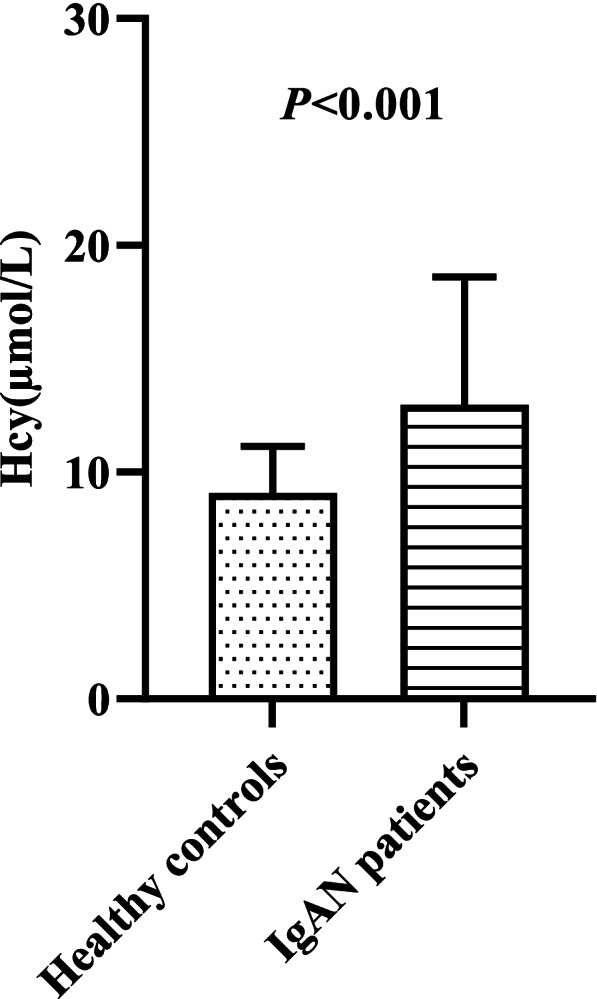
Comparison of serum Hcy between healthy controls and IgAN patients

**Fig. 2:**
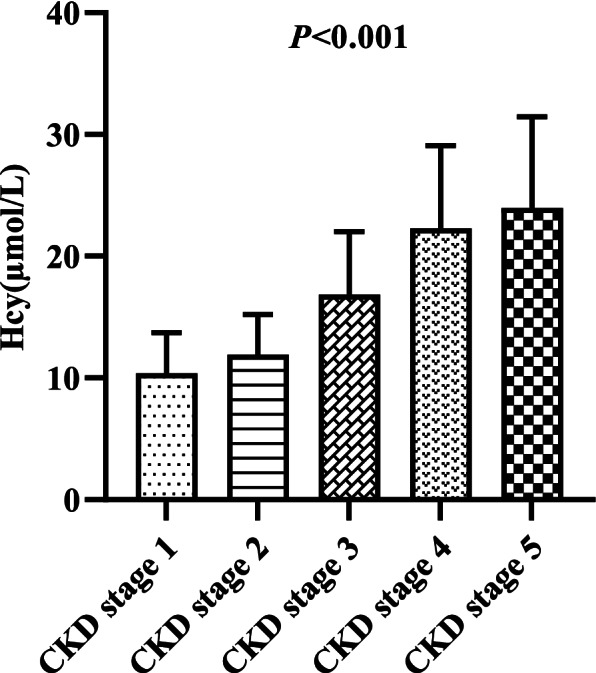
Serum levels of Hcy in different CKD stage among IgAN patients

**Fig. 3:**
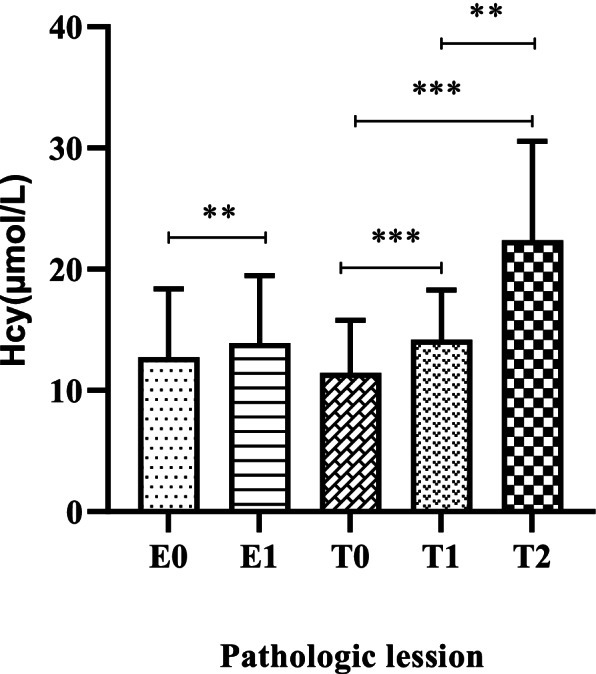
Comparison of serum Hcy in lesions of endocapillary hypercellularity (E) and tubular atrophy/interstitial fibrosis (T) among IgAN patients.**P < 0.05, ***P < 0.001

Serum levels of Hcy [11.6 (9.1, 15.3) vs. 8.8 (7.5, 10.6) μmol/L, *P* < 0.001; Fig. 1] were significantly higher in the IgAN group than in the healthy control group. A total of 156 patients had normal renal function with eGFR ≥90 ml/min/1.73 m^2^, and 74 patients were in the high Hcy group. The median level of Hcy was 9.9 (7.6, 12.4) μmol/L in CKD stage 1 patients, which was still higher than that in healthy controls (*P* < 0.001). The median Hcy levesl of CKD stage 1-5 patients were 9.9 (7.6, 12.4), 11.1 (9.6, 13.7), 15.7 (12.9, 18.8), 24.4 (16.1, 27.0), and 23.8 (16.9, 31.7) μmol/L, respectively (*P* < 0.001; Fig. 2). Hcy was 13.5 (10.5, 17.5) μmol/L and 10.7 (8.1, 13.6) μmol/L in male and female patients (*P* < 0.001), respectively. Compared with patients without endocapillary hypercellularity (E0), those with endocapillary hypercellularity (E1) had significantly elevated Hcy [13.2 (10.4, 15.8) vs. 11.2 (8.9, 14.9) μmol/L, *P* = 0.027; Fig. 3]. Patients with more severe tubular atrophy/interstitial fibrosis lesions showed significantly higher serum levels of Hcy [T_2_: 22.7 (15.7, 30.0) vs. T_1_: 13.6 (11.2, 15.7) vs. T_0_: 10.5 (8.3, 13.4) μmol/L, *P* < 0.001; Fig. 3].

### Comparison of clinicopathologic characteristics between the low Hcy group and the high Hcy group

A total of 114 patients were in the low Hcy group and 223 patients were in the high Hcy group. The clinical characteristics and histopathological features are presented in Table 1. Compared with the low Hcy group, patients in the high Hcy group were older (*P =* 0.003), had a higher proportion of male patients (*P <* 0.001) and had a history of HBP (*P <* 0.001). Significantly higher levels of MAP (*P <* 0.001), Scr (*P <* 0.001), BUN (*P <* 0.001), UA (*P <* 0.001), ALB (*P =* 0.011), and hsCRP (*P =* 0.033) and lower levels of eGFR (*P <* 0.001), HDL (*P <* 0.001), and IgM (*P =* 0.024) were shown in the high Hcy group. Pathological E (*P =* 0.041) and T (*P <* 0.001) lesions were significantly more serious in the elevated Hcy group than in the low Hcy group. Difference in 24-h urine protein, Hb and complement were not discovered between the two groups.

**Table 1:** Clinicopathologic characteristics of IgAN patients in low and high Hcy group

Variable	Total (*n* = 337)	Hcy ≤ 10.0 (*n* = 114)	Hcy > 10.0 (*n* = 223)	*P*
Hcy (μmol/L)	11.6 (9.1,15.3)	8.2 (7.3,9.1)	13.7 (11.7,16.7)	< 0.001^**^
Age (years)	34.0 (27.5,44.0)	32.5 (27.0,39.0)	35.0 (28.0,47.0)	0.003^*^
Male, n (%)	135 (40.1)	27 (23.7)	108 (48.4)	< 0.001^**^
SBP (mmHg)	123.0 (115.0,137.5)	119.5 (110.0,131.0)	126.0 (117.0,141.0)	< 0.001^**^
DBP (mmHg)	80.0 (74.0,90.0)	77.5 (73.0,87.0)	82.0 (75.0,94.0)	0.002^*^
MAP (mmHg)	94.7 (88.7,106.3)	92.0 (86.3,99.7)	96.3 (89.3,109.7)	< 0.001^**^
HBP, n (%)	132 (39.2)	22 (19.3)	110 (49.3)	< 0.001^**^
Scr (μmol/L)	86.0 (70.0,115.5)	69.5 (61.0,82.3)	99.0 (77.0,139.0)	< 0.001^**^
eGFR (mL/min/1.73 m^2^)	88.1 (62.8,103.4)	101.4 (89.3,115.4)	75.0 (44.9,95.2)	< 0.001^**^
CKD stage, n (%)				< 0.001^**^
CKD 1	156 (46.3)	82 (71.9)	74 (33.2)	
CKD 2	100 (29.7)	32 (28.1)	68 (30.5)	
CKD 3	51 (15.1)	0 (0.0)	51 (22.9)	
CKD 4	13 (3.9)	0 (0.0)	13 (5.8)	
CKD 5	17 (5.0)	0 (0.0)	17 (7.6)	
BUN (mmol/L)	5.1 (4.3,6.9)	4.5 (3.6,5.1)	5.8 (4.7,8.0)	< 0.001^**^
UA (mmol/L)	390.0 (324.0,484.5)	352.5 (302.8,413.8)	413.0 (347.0,512.0)	< 0.001^**^
24-h urine protein (g/24 h)	0.6 (0.2,1.7)	0.5 (0.2,1.0)	0.7 (0.3,1.8)	0.101
Hb (g/L)	125.0 (114.0,139.5)	128.5 (118.8,138.0)	124.0 (111.0,142.0)	0.135
ALB (g/L)	35.0 ± 6.8	33.7 ± 7.9	35.6 ± 6.2	0.011^*^
Glu (mmol/L)	4.4 ± 0.6	4.4 ± 0.5	4.4 ± 0.6	0.631
CHOL (mmol/L)	5.1 (4.3,5.9)	5.1 (4.5,6.0)	5.0 (4.3,5.9)	0.262
TG (mmol/L)	1.6 ± 1.4	1.4 ± 0.9	1.7 ± 1.6	0.080
LDL (mmol/L)	3.2 (2.6,3.8)	3.2 (2.7,3.9)	3.2 (2.6,3.8)	0.386
HDL (mmol/L)	1.3 ± 0.4	1.4 ± 0.4	1.2 ± 0.3	< 0.001^**^
SOD (U/ml)	136.0 ± 26.6	138.4 ± 30.3	134.8 ± 24.5	0.241
hsCRP (mg/L)	0.7 (0.3,1.8)	0.5 (0.3,1.5)	0.8 (0.3,2.0)	0.033^*^
ESR (mm/h)	17.0 (9.0,32.0)	16.5 (10.0,27.0)	19.0 (8.0,35.0)	0.647
IgA (g/L)	3.3 ± 1.1	3.3 ± 1.1	3.3 ± 1.2	0.695
IgM (g/L)	1.2 ± 0.6	1.3 ± 0.6	1.2 ± 0.5	0.024^*^
IgG (g/L)	11.5 ± 3.3	11.0 ± 3.5	11.7 ± 3.2	0.066
C3 (mg/L)	1054.2 ± 202.2	1077.9 ± 213.5	1042.0 ± 195.7	0.125
C4 (mg/L)	258.4 ± 91.6	250.9 ± 91.1	262.2 ± 91.9	0.288
Oxford classification, n (%)				
M1	320 (95.0)	108 (94.7)	212 (95.1)	0.896
E1	65 (19.3)	15 (13.2)	50 (22.4)	0.041^*^
S1	132 (39.2)	42 (36.8)	90 (40.4)	0.531
T				< 0.001^**^
T0	238 (70.6)	106 (93.0)	132 (59.2)	
T1	70 (20.8)	7 (6.1)	63 (28.3)	
T2	29 (8.6)	1 (0.9)	28 (12.6)	
C				0.524
C0	150 (44.5)	54 (47.4)	96 (43.0)	
C1	166 (49.3)	55 (48.2)	111 (49.8)	
C2	21 (6.2)	5 (4.4)	16 (7.2)	

*Abbreviations*: *Hcy* homocysteine, *SBP* systolic blood pressure, *DBP* diastolic blood pressure, *MAP* mean arterial pressure, *HBP* Hypertension, *Scr* serum creatinine, *eGFR* estimated glomerular filtration rate, *CKD* chronic kidney disease, *BUN* blood urine nitrogen, *UA* uric acid, *Hb* hemoglobin, *ALB* serum albumin, *Glu* fasting glucose, *CHOL* cholesterol, *TG* triglyceride, *LDL* low-density lipoprotein-cholesterol, *HDL* high-density lipoprotein-cholesterol, *SOD* superoxide dismutase, *hsCRP* high sensitivity C reaction protein, *ESR* erythrocyte sedimentatio rate, *IgA* immunoglobulin A, *IgM* immunoglobulin M, *IgG* immunoglobulin G, *C3* complement3, *C4* complement4, *M* mesangial hypercellularity, *E* endocapillary hypercellularity, *S* segmental glomerulosclerosis, *T* tubular atrophy and interstitial fibrosis, *C* crescents

*P < 0.05, **P < 0.001

### Correlations between Hcy and clinicopathologic parameters

Hcy levels were positively correlated with age (*r* = 0.18, *P* = 0.001), MAP (*r* = 0.24, *P* < 0.001), Scr (*r* = 0.69, *P* < 0.001), BUN (*r* = 0.57, *P* < 0.001), UA (*r* = 0.42, *P* < 0.001), 24-h urine protein (*r* = 0.22, *P* < 0.001), TG (*r* = 0.21, *P* < 0.001), hsCRP (*r* = 0.22, *P* < 0.001), ESR (*r* = 0.13, *P* = 0.016), C4 (*r* = 0.14, *P* = 0.010), pathologic E (*r* = 0.12, *P* = 0.026), and pathologic T (*r* = 0.45, *P* < 0.001), but negatively correlated with eGFR (*r* = − 0.62, *P* < 0.001), Hb (*r* = − 0.15, *P* = 0.008), HDL (*r* = − 0.31, *P* < 0.001), SOD (*r* = − 0.23, *P* < 0.001), and IgM (*r* = − 0.16, *P* = 0.004). However, relationships between Hcy and ALB, Glu, CHOL, LDL, IgA, IgG, C3, and pathologic M, S, and C were not found (*P* > 0.05). The correlations between Hcy and the clinicopathological features of IgAN patients are shown in Table 2.

**Table 2:** Correlations between Hcy and clinicopathologic parameters in IgAN patients

Variable	*r*	*P*
Age	0.18	0.001^*^
MAP	0.24	< 0.001^**^
Scr	0.69	< 0.001^**^
eGFR	−0.62	< 0.001^**^
BUN	0.57	< 0.001^**^
UA	0.42	< 0.001^**^
24-h urine protein	0.22	< 0.001^**^
Hb	−0.15	0.008^*^
ALB	0.05	0.338
Glu	0.02	0.695
CHOL	−0.02	0.718
TG	0.21	< 0.001^**^
LDL	0.01	0.834
HDL	−0.31	< 0.001^**^
SOD	−0.23	< 0.001^**^
hsCRP	0.22	< 0.001^**^
ESR	0.13	0.016^*^
IgA	0.04	0.435
IgM	−0.16	0.004^*^
IgG	0.07	0.214
C3	0.03	0.533
C4	0.14	0.010^*^
M	0.01	0.869
E	0.12	0.026^*^
S	0.04	0.500
T	0.45	< 0.001^**^
C	−0.01	0.822

*Abbreviations*: *MAP* mean arterial pressure, *Scr* serum creatinine, *eGFR* estimated glomerular filtration rate, *BUN* blood urine nitrogen, *UA* uric acid, *Hb* hemoglobin, *ALB* serum albumin, *Glu* fasting glucose, *CHOL* cholesterol, *TG* triglyceride, *LDL* low-density lipoprotein-cholesterol, *HDL* high-density lipoprotein-cholesterol, *SOD* superoxide dismutase, *hsCRP* high sensitivity C reaction protein, *ESR* erythrocyte sedimentatio rate, *IgA* immunoglobulin A, *IgM* immunoglobulin M, *IgG* immunoglobulin G, *C3* complement3, *C4* complement4, *M* mesangial hypercellularity, *E* endocapillary hypercellularity, *S* segmental glomerulosclerosis, *T* tubular atrophy and interstitial fibrosis, *C* crescents

*P < 0.05, **P < 0.001

### Risk factor analysis of IgAN associated with elevated Hcy

The logistic regression analysis results are shown in Table 3. Univariate analysis revealed that IgAN patients who had lower levels of HDL and IgM, and higher levels of age, MAP, Scr, BUN, UA, ALB, ESR, and pathologic E and T lesions were at a greater risk of elevated Hcy. After the variable selection procedure, the final multivariate logistic regression model showed that male sex [odds ratio (OR) = 3.47, 95% confidence interval (CI) = 1.86-6.48, *P* < 0.001)], lower eGFR (OR = 0.95, 95% CI = 0.94-0.97, *P* < 0.001), higher ALB (OR = 1.07, 95% CI = 1.03-1.12, *P* = 0.001), and pathologic T (OR = 2.87, 95% CI = 1.20-6.89, *P* = 0.018) independently increased the risk of elevated Hcy in IgAN patients.

**Table 3:** Clinicopathologic features as factors of elevated Hcy by univariate and multivariate logistic regression analysis

Variables	Univariate		Multivariate			
			Overall		eGFR≥90 ml/min/1.73 m^2^	
	Odds ratio (95% CI)	*P*	Odds ratio (95% CI)	*P*	Odds ratio (95%CI)	*P*
Age	1.04 (1.02-1.06)	< 0.001^**^				
Male	3.03 (1.83-5.02)	< 0.001^**^	3.47 (1.86-6.48)	< 0.001^**^	5.31 (2.23-12.64)	< 0.001^**^
MAP	1.04 (1.02-1.05)	< 0.001^**^				
Scr	1.07 (1.05-1.09)	< 0.001^**^				
eGFR	0.95 (0.94-0.96)	< 0.001^**^	0.95 (0.94-0.97)	< 0.001^**^	0.95 (0.91-0.98)	0.003^*^
BUN	1.66 (1.40-1.98)	< 0.001^**^				
UA	1.01 (1.00-1.01)	< 0.001^**^				
24-h urine protein	1.00 (0.94-1.08)	0.929				
Hb	0.99 (0.98-1.00)	0.085				
ALB	1.04 (1.01-1.08)	0.013^*^	1.07 (1.03-1.12)	0.001^*^	1.10 (1.04-1.16)	0.001^*^
Glu	0.91 (0.60-1.36)	0.630				
CHOL	0.87 (0.77-1.00)	0.042^*^				
TG	1.24 (0.98-1.58)	0.079				
LDL	0.83 (0.69-1.01)	0.061				
HDL	0.31 (0.16-0.61)	0.001^*^				
SOD	1.00 (0.99-1.00)	0.241				
hsCRP	1.01 (0.98-1.04)	0.429				
ESR	1.01 (1.00-1.02)	0.033^*^				
Ig A	1.04 (0.85-1.28)	0.694				
Ig M	0.63 (0.42-0.95)	0.027^*^				
Ig G	1.07 (1.00-1.14)	0.068				
C3	1.00 (1.00-1.00)	0.126				
C4	1.00 (1.00-1.00)	0.288				
M0	1 (reference)					
M1	1.07 (0.39-2.97)	0.896				
E0	1 (reference)					
E1	1.91 (1.02-3.57)	0.044^*^				
S0	1 (reference)					
S1	1.16 (0.73-1.85)	0.532				
T0	1 (reference)		1 (reference)		1 (reference)	
T1-2	9.13 (4.24-19.66)	< 0.001^**^	2.87 (1.20-6.89)	0.018^*^	14.20 (1.68-120.03)	0.015^*^
C0	1 (reference)					
C1-2	1.19 (0.76-1.87)	0.451				

*Abbreviations*: *MAP* mean arterial pressure, *Scr* serum creatinine, *eGFR* estimated glomerular filtration rate, *BUN* blood urine nitrogen, *UA* uric acid, *Hb* hemoglobin, *ALB* serum albumin, *Glu* fasting glucose, *CHOL* cholesterol, *TG* triglyceride, *LDL* low-density lipoprotein-cholesterol, *HDL* high-density lipoprotein-cholesterol, *SOD* superoxide dismutase, *hsCRP* high sensitivity C reaction protein, *ESR* erythrocyte sedimentatio rate, *IgA* immunoglobulin A, *IgM* immunoglobulin M, *IgG* immunoglobulin G, *C3* complement3, *C4* complement4, *M* mesangial hypercellularity, *E* endocapillary hypercellularity, *S* segmental glomerulosclerosis, *T* tubular atrophy and interstitial fibrosis, *C* crescents

*P < 0.05, **P < 0.001

### Subgroup analysis in IgAN patients with normal renal function

A total of 156 patients had normal renal function with eGFR ≥90 ml/min/1.73 m^2^. Seventy-four patients were in the high Hcy group, with a higher proportion of male patients, and a history of HBP, SBP, Scr, BUN, ALB, and pathological T lesions and a lower eGFR and 24-h urine protein than those in the low Hcy group. The results of the subgroup analysis are presented in Supplement Table 1. As shown in Supplement Table 2, Hcy levels were positively correlated with Scr, BUN, UA, ALB and T lesions, but negatively correlated with eGFR and HDL. Moreover, multivariate logistic regression analysis adjusted by male sex, eGFR, and ALB also showed that pathologic T was more related to elevated Hcy (OR = 14.20, 95% CI = 1.68-120.03, *P* = 0.015) in the subgroup of patients with eGFR ≥90 ml/min/1.73 m^2^.

## Discussion

In this study, we found that the serum Hcy of IgAN patients was significantly higher than that of healthy controls in our center. IgAN patients with elevated Hcy displayed more severe T lesions, even in subgroups of patients with normal renal function. Multivariate logistic regression analysis showed that pathologic T was an independent risk factor for elevated Hcy in IgAN patients and persisted in a subgroup analysis according to eGFR ≥90 ml/min/1.73 m^2^.

In this study, we found that Hcy was positively correlated with age in the whole cohort of 337 IgAN patients. A serious shortage of vitamin B12 and vitamin B6 was common in elderly populations, and a lack of cofactors reduced the metabolism of Hcy. In addition, the decline in renal function with aging also caused elevation of Hcy [14]. Significantly higher serum levels of Hcy were observed in male patients with IgAN than that in female patients [13.5 (10.5, 17.5) vs. 10.7 (8.1, 13.6) μmol/L, *P* < 0.001], which was similar to a previous study [15, 16]. Moreover, males were independently associated with elevated Hcy in our study. One possible reason was the difference between the sexes in lifestyle variables that are known to have an impact on Hcy [17]. In addition, estrogen could decrease plasma Hcy levels [18]. As previously reported [19], patients with elevated Hcy had higher ALB levels. The mechanism was not well elucidated. Scholars have explained that Hcy covalently binds to ALB, resulting in the lack of filtration and absorption processes of Hcy in the kidney [20]. However, more studies are needed to probe the link between ALB and Hcy and its underlying mechanism.

From the results in this study, it can be seen that serum Hcy was closely related to kidney function. A previous study demonstrated that serum Hcy was very likely elevated in patients with CKD and gradually increased with the progression of CKD stage [4]. Hcy was markedly elevated at the ESRD stage [21], and was 3-5 times that of healthy participants [22]. In our study, the serum Hcy of IgAN patients was similarly elevated compared to that of sex- and age- matched healthy controls, even in a subgroup of patients with normal renal function, and increased with the decline in eGFR. However, there was a question as to whether the elevated Hcy was a cause for CKD or rather the result of CKD.

It has been demonstrated that the kidney is a major site for the removal of plasma Hcy and plays an important role in the regulation of plasma Hcy levels [23]. In our study, multivariate logistic regression analysis found that pathologic T (OR = 2.87, 95% CI = 1.20-6.89, *P* = 0.018) was an independent risk factor for elevated Hcy in IgAN patients, even after adjustment for eGFR and in the subgroup with normal renal function (OR = 14.20, 95% CI = 1.68-120.03, *P* = 0.015). Zhang et al. observed the association between Hcy and pathologic T lesions in IgAN [11]. Given impairment in remethylation, patients with CKD were likely more dependent on the pathway of Hcy transsulfuration, which was catalyzed by cystathione-β-synthase (CBS) and γ-cystathionase (CTL). Meanwhile, James D et al. expressed that CBS was enriched in the proximal convoluted tubule cells. CTLs exhibit higher enrichment patterns in proximal straight tubule cells [24]. Li et al. found that renal proximal tubules were major sites for Hcy metabolism in the kidney [25]. When tubular atrophy and interstitial fibrosis occurr, the transsulfuration pathway is reduced, leading to the elevation of Hcy. It seemed that the increase in Hcy was the result of renal injury, especially T lesions.

The Oxford classification score is a well-regarded prognostic indicator for IgAN. Tubular interstitial lesions are the most valuable histological parameter and a final pathway for most progressive kidney diseases. T lesions are common in IgAN patients even with normal renal function; however, factors associated with T lesions in IgAN have not been elucidated. Possible mechanisms to explain the T lesions are that with aggravating renal impairment, renal units will be damaged accompanied by fibrosis, resulting in loss of normal excretory function and reduced clearance of Hcy. On the other hand, IgAN is an inflammatory disease, and Hcy is related to the inflammatory response and stimulates the production of cytokines and proinflammatory molecules, which may be related to renal fibrosis. We found that nearly half of IgAN patients had elevated Hcy levels and displayed more severe clinicopathologic characteristics, even in patients with normal renal function with eGFR ≥90 ml/min/1.73 m^2^. A longitudinal prospective study showed that elevated plasma Hcy levels may be a predictor of accelerated decline in renal function and future incidence of CKD [6]. An animal study implicated elevated Hcy related to glomerular injury [26]. In both essential hypertension and general adult populations, high Hcy was associated with albuminuria independent of renal function [27, 28]. A prospective study showed that Hcy was an independent determinant of the development of albuminuria even after adjustment for eGFR [29]. In fact, Hcy is associated with vascular damage, endothelial dysfunction and cell proliferation, oxidative stress, lipid and lipoprotein metabolism and inflammation [30].

In addition, serum Hcy was positively correlated with hsCRP and ESR, but negatively correlated with SOD in the whole cohort of IgAN patients. Gori et al. revealed that interleukin-6 and interleukin-1 were independent predictors of Hcy concentrations in elderly individuals [31]. Decreased SOD activity was observed in the kidneys of high Hcy rats [32]. SOD inhibits antioxidants and catalyzes superoxide conversion to H_2_O_2_. Hcy directly inhibits the activity of antioxidants, thereby disrupting SOD. On the other hand, elevated Hcy could induce oxidative stress, resulting in excessive superoxide production and SOD consumption [33]. The clinical results supported the findings of animal and cell model studies that Hcy promoted inflammation, redox imbalance and oxidative stress [34], which contributed to renal vascular damage. As a consequence, high Hcy levels may potentially induce renal injury and not only be the result of impaired renal function.

Of note, we investigated the relationship between Hcy and clinicopathologic characteristics in the whole cohort of 337 IgAN patients and then in the subgroup with eGFR ≥90 ml/min/1.73 m^2^ to modify the effect of renal impairment. Our study showed consistent results that higher Hcy was associated with higher Scr, BUN, UA, ALB and T lesions but lower eGFR and HDL. However, there were opposite results in the correlation between Hcy and age, 24-h urine protein, and ESR at the early stage of 156 IgAN patients, which might be partially explained by the small sample size.

To date, whether Hcy is just a marker or plays a causal role in IgAN remains to be elucidated. The main strength of this study was that we assessed the association between serum Hcy and clinicopathologic characteristics in IgAN patients with normal renal function. We observed that elevated Hcy displayed more severe clinicopathologic characteristics, and pathologic T was an independent risk factor for elevated Hcy even in the early stage of IgAN. Our study prompted clinicians to pay more attention to Hcy levels in IgAN patients, especially at early stages. Previously, a randomized controlled trial in China suggested that Hcy-lowering therapy can significantly delay the progression of renal impairment among patients with mild-to-moderate CKD [35]. Further studies are needed in the future to discover the controversial relationship between kidney function and Hcy and confirm whether Hcy concentration intervention is beneficial to IgAN patients.

The present study had some limitations. First, because of the cross-sectional design of this study, we cannot obtain any causal inferences from the data. Second, as a single-center study, our study could not exclude the limits of races and regions, and its external validity may be limited. Third, the serum Hcy cutoff points differed among the previous studies [4, 11, 36]. However, Chinese expert consensus on hyperhomocysteinemia published in 2020 defined hyperhomocysteinemia as Hcy levels greater than 10 μmol/L. A study of Hcy and Chinese patients with IgA nephropathy also defined hyperhomocysteinemia as Hcy levels greater than 10 μmol/L [11]. The population in our study was from China, so 10 μmol/L was selected as the Hcy cutoff in this study. Fourth, Hcy may be affected by folate, vitamin B12 and vitamin B6 status, which were not assessed in this study. In addition, there is a lack of follow-up data to assess the impact of baseline serum Hcy on renal and CVD outcomes in patients with IgAN.

## Conclusions

IgAN patients with elevated serum Hcy displayed more severe clinicopathologic characteristics. Pathologic T was an independent risk factor associated with elevated Hcy in IgAN patients, especially at the early stage of IgAN.

## Supplementary Information

**Additional file 1: Supplement Table 1.** Clinicopathologic characteristics comparison in the subgroup of IgAN patients with eGFR≥90 ml/min/1.73 m^2^. **Supplement Table 2.** Correlations between Hcy and clinicopathologic parameters in the subgroup of IgAN patients with eGFR≥90 ml/min/1.73 m^2^. **Supplement Table 3.** Clinicopathologic features as factors of elevated Hcy by univariate and multivariate logistic regression analysis in whole populations with "Enter" method. **Supplement Table 4.** Clinicopathologic features as factors of elevated Hcy by univariate and multivariate logistic regression analysis in patients with eGFR≥90 ml/min/1.73 m^2^ with "Enter" method.

## Data Availability

The datasets used and/or analyzed during the current study available from the corresponding author on reasonable request.

## Abbreviations

Hcy: Homocysteine
CVD: Cardiovascular disease
CKD: Chronic kidney disease
IgAN: IgA nephropathy
ESRD: End-stage renal disease
MR: Mendelian randomization
BP: Blood pressure
ANCA: Anti-neutrophil cytoplasm antibody
SBP: Systolic blood pressure
DBP: Diastolic blood pressure
MAP: Mean arterial pressure
HBP: Hypertension
Scr: Serum creatinine
eGFR: Estimated glomerular filtration rate
BUN: Blood urine nitrogen
UA: Uric acid
Hb: Hemoglobin
ALB: Serum albumin
Glu: Fasting glucose
CHOL: Cholesterol
TG: Triglyceride
LDL: Low-density lipoprotein-cholesterol
HDL: High-density lipoprotein-cholesterol
SOD: Superoxide dismutase
hsCRP: High sensitivity C reaction protein
ESR: Erythrocyte sedimentatio rate
IgA: Immunoglobulin A
IgM: Immunoglobulin M
IgG: Immunoglobulin G
C3: Complement3
C4: Complement4
CKD-EPI: Chronic Kidney Disease Epidemiology Collaboration
M: Mesangial hypercellularity
E: Endocapillary hypercellularity
S: Segmental glomerulosclerosis
T: Tubular atrophy and interstitial fibrosis
C: Crescents
CBS: Cystathione-β-synthase
CTL: γ-cystathionase
OR: Odds ratio
95% CI: 95% Confidence interval
