# Supplementary material for: Serum homocysteine is associated with tubular interstitial lesions at the early stage of IgA nephropathy
Source: BMC Nephrol. 2022 Feb 23;23:78. doi: 10.1186/s12882-021-02632-3 (PMC8867621; doi:10.1186/s12882-021-02632-3)
Supplement: Supplementary file 1 — Additional file 1: Supplement Table 1. Clinicopathologic characteristics comparison in the subgroup of IgAN patients with eGFR≥90 ml/min/1.73 m2. Supplement Table 2. Correlations between Hcy and clinicopathologic parameters in the subgroup of IgAN patients with eGFR≥90 ml/min/1.73 m2. Supplement Table 3. Clinicopathologic features as factors of elevated Hcy by univariate and multivariate logistic regression analysis in whole populations with "Enter" method. Supplement Table 4. Clinicopathologic features as factors of elevated Hcy by univariate and multivariate logistic regression analysis in patients with eGFR≥90 ml/min/1.73 m2 with "Enter" method. [file 12882_2021_2632_MOESM1_ESM.doc]

**Supplement Table1.** Clinicopathologic characteristics comparison in the subgroup of IgAN patients with eGFR≥90 ml/min/1.73 m2

| Variable | Total (n=156) | Hcy≤10.0 (n=82) | Hcy>10.0 (n=74) | *P* |
| --- | --- | --- | --- | --- |
| Hcy (µmol/L) | 9.9(7.6,12.4) | 7.7(7.1,8.9) | 12.6(11.0,14.8) | <0.001^**^ |
| Age (years) | 31.1±8.6 | 31.9±8.9 | 30.2±8.2 | 0.196 |
| Male, n (%) | 53(34.0) | 17(20.7) | 36(48.6) | <0.001^**^ |
| SBP (mmHg ) | 120.5±15.0 | 118.2±13.1 | 123.1±16.5 | 0.039^*^ |
| DBP ( mmHg) | 78.9±10.6 | 78.0±9.2 | 79.8±12.0 | 0.297 |
| MAP (mmHg) | 92.7±11.2 | 91.4±10.1 | 94.2±12.3 | 0.116 |
| HBP, n (%) | 33(21.2) | 10(12.2) | 23(31.1) | 0.004^*^ |
| Scr(µmol/L) | 69.0(62.3,78.8) | 64.0(60.0,71.3) | 74.0(68.0,88.0) | <0.001^**^ |
| eGFR (mL/min/1.73 m^2^) | 105.3(98.1,225.2) | 111.5(99.9,118.5) | 101.4(95.1,110.0) | <0.001^**^ |
| BUN (mmol/L) | 4.5±1.2 | 4.3±1.3 | 4.8±1.1 | 0.011^*^ |
| UA(mmol/L) | 364.6±98.0 | 353.3±90.1 | 377.2±105.2 | 0.129 |
| 24-h urine protein (g/24 h) | 0.4(0.2,0.8) | 0.5(0.2,1.1) | 0.3(0.1,0.7) | 0.043^*^ |
| Hb (g/L) | 130.5±18.1 | 128.2±18.5 | 133.2±17.5 | 0.085 |
| ALB (g/L) | 36.9(33.2,40.1) | 35.2(32.1,38.4) | 38.5(35.0,41.4) | <0.001^**^ |
| Glu (mmol/L) | 4.4±0.5 | 4.4±0.5 | 4.3±0.5 | 0.324 |
| CHOL(mmol/L) | 4.9(4.2,5.7) | 5.0(4.3,6.1) | 4.9(4.2,5.5) | 0.263 |
| TG (mmol/L) | 1.3±0.7 | 1.3±0.7 | 1.3±0.7 | 0.955 |
| LDL (mmol/L) | 3.1(2.5,3.7) | 3.2(2.6,3.8) | 3.1(2.5,3.7) | 0.406 |
| HDL (mmol/L) | 1.3±0.4 | 1.4±0.4 | 1.3±0.3 | 0.052 |
| SOD (U/ml) | 140.0±28.9 | 137.0±31.8 | 143.2±25.3 | 0.180 |
| hsCRP (mg/L) | 0.5(0.3,1.2) | 0.5(0.3,1.2) | 0.5(0.3,1.1) | 0.678 |
| ESR (mm/h) | 18.9±16.6 | 22.0±17.3 | 15.5±15.1 | 0.014^*^ |
| IgA (g/L) | 3.3±0.9 | 3.3±1.0 | 3.2±0.9 | 0.443 |
| IgM (g/L) | 1.3±0.6 | 1.3±0.6 | 1.2±0.6 | 0.274 |
| IgG (g/L) | 11.3±3.4 | 11.0±3.6 | 11.6±3.1 | 0.284 |
| C3 (mg/L) | 1035.1±197.5 | 1062.9±214.3 | 1004.7±173.8 | 0.067 |
| C4 (mg/L) | 235.3±83.6 | 241.3±94.7 | 228.8±69.5 | 0.356 |
| Oxford classification, n (%) |  |  |  |  |
| M1 | 145(92.9) | 78(95.1) | 67(90.5) | 0.264 |
| E1 | 22(14.1) | 11(13.4) | 11(14.9) | 0.795 |
| S1 | 45(28.8) | 28(34.1) | 17(23.0) | 0.124 |
| T |  |  |  | 0.001^*^ |
| T0 | 143(91.7) | 81(98.8) | 62(83.8) |  |
| T1 | 13(8.3) | 1(1.2) | 12(16.2) |  |
| T2 | 0 | 0 | 0 |  |
| C |  |  |  | 0.358 |
| C0 | 70(44.9) | 40(48.8) | 30(40.5) |  |
| C1 | 77(49.4) | 39(47.6) | 38(51.4) |  |
| C2 | 9(5.8) | 3(3.7) | 6(8.1) |  |

Abbreviations: Hcy, homocysteine; SBP, systolic blood pressure; DBP, diastolic blood pressure; MAP, mean arterial pressure; HBP, Hypertension; Scr, serum creatinine; eGFR, estimated glomerular filtration rate; BUN, blood urine nitrogen;UA,uric acid; Hb, hemoglobin; ALB, serum albumin; Glu, fasting glucose; CHOL, cholesterol; TG, triglyceride; LDL, low-density lipoprotein-cholesterol; HDL, high-density lipoprotein-cholesterol; SOD, superoxide dismutase; hsCRP, high sensitivity C reaction protein; ESR, erythrocyte sedimentatio rate; IgA, immunoglobulin A; IgM, immunoglobulin M; IgG, immunoglobulin G; C3, complement3; C4, complement4; M, mesangial hypercellularity; E, endocapillary hypercellularity; S, segmental glomerulosclerosis; T, tubular atrophy and interstitial fibrosis; C, crescents.

*P< 0.05, **P< 0.001.

**Supplement Table 2.** Correlations between Hcy and clinicopathologic parameters in the subgroup of IgAN patients with eGFR≥90 ml/min/1.73 m2

| Variable | *r* | *P* |
| --- | --- | --- |
| Age | -0.25 | 0.001^*^ |
| MAP | 0.04 | 0.656 |
| Scr | 0.51 | <0.001^**^ |
| eGFR | -0.26 | <0.001^**^ |
| BUN | 0.30 | <0.001^**^ |
| UA | 0.21 | 0.009^*^ |
| 24-h urine protein | -0.12 | 0.139 |
| Hb | 0.15 | 0.060 |
| ALB | 0.35 | <0.001^**^ |
| Glu | -0.04 | 0.618 |
| CHOL | -0.07 | 0.379 |
| TG | 0.01 | 0.947 |
| LDL | -0.05 | 0.555 |
| HDL | -0.18 | 0.026^*^ |
| SOD | 0.06 | 0.495 |
| hsCRP | -0.01 | 0.990 |
| ESR | -0.28 | <0.001^**^ |
| IgA | -0.03 | 0.755 |
| IgM | -0.08 | 0.308 |
| IgG | 0.05 | 0.509 |
| C3 | -0.06 | 0.474 |
| C4 | 0.02 | 0.982 |
| M | -0.10 | 0.223 |
| E | 0.06 | 0.430 |
| S | -0.17 | 0.039^*^ |
| T | 0.25 | 0.002^*^ |
| C | 0.04 | 0.626 |

Abbreviations: MAP, mean arterial pressure; Scr, serum creatinine; eGFR, estimated glomerular filtration rate; BUN, blood urine nitrogen;UA,uric acid; Hb, hemoglobin; ALB, serum albumin; Glu, fasting glucose; CHOL, cholesterol; TG, triglyceride; LDL, low-density lipoprotein-cholesterol; HDL, high-density lipoprotein-cholesterol; SOD, superoxide dismutase; hsCRP, high sensitivity C reaction protein; ESR, erythrocyte sedimentatio rate; IgA, immunoglobulin A; IgM, immunoglobulin M; IgG, immunoglobulin G; C3, complement3; C4, complement4; M, mesangial hypercellularity; E, endocapillary hypercellularity; S, segmental glomerulosclerosis; T, tubular atrophy and interstitial fibrosis; C, crescents.

*P< 0.05, **P< 0.001.

**Supplement Table 3.** Clinicopathologic features as factors of elevated Hcy by univariate and multivariate logistic regression analysis in whole populations

| Variables | univariate |  | multivariate |  |
| --- | --- | --- | --- | --- |
|  | Odds ratio (95% CI) | *P* | Odds ratio (95% CI) | *P* |
| Age | 1.04(1.02-1.06) | <0.001^**^ | 0.99(0.96-1.03) | 0.720 |
| Male | 3.03(1.83-5.02) | <0.001^**^ | 3.99(1.68-9.48) | 0.002^*^ |
| MAP | 1.04(1.02-1.05) | <0.001^**^ | 1.00(0.97-1.03) | 0.895 |
| Scr | 1.07(1.05-1.09) | <0.001^**^ |  |  |
| eGFR | 0.95(0.94-0.96) | <0.001^**^ | 0.95(0.93-0.98) | <0.001^**^ |
| BUN | 1.66(1.40-1.98) | <0.001^**^ | 1.18(0.95-1.46) | 0.145 |
| UA | 1.01(1.00-1.01) | <0.001^**^ | 1.00(1.00-1.00) | 0.831 |
| 24-h urine protein | 1.00(0.94-1.08) | 0.929 |  |  |
| Hb | 0.99(0.98-1.00) | 0.085 | 1.00(0.98-1.02) | 0.915 |
| ALB | 1.04(1.01-1.08) | 0.013^*^ | 1.07(1.01-1.14) | 0.028^*^ |
| Glu | 0.91(0.60-1.36) | 0.630 |  |  |
| CHOL | 0.87(0.77-1.00) | 0.042^*^ | 1.18(0.34-1.42) | 0.798 |
| TG | 1.24(0.98-1.58) | 0.079 | 0.77(0.52-1.13) | 0.179 |
| LDL | 0.83(0.69-1.01) | 0.061 | 0.61(0.13-2.87) | 0.531 |
| HDL | 0.31(0.16-0.61) | 0.001^*^ | 0.98(0.18-5.26) | 0.981 |
| SOD | 1.00(0.99-1.00) | 0.241 |  |  |
| hsCRP | 1.01(0.98-1.04) | 0.429 |  |  |
| ESR | 1.01(1.00-1.02) | 0.033^*^ | 1.01(0.99-1.03) | 0.443 |
| Ig A | 1.04(0.85-1.28) | 0.694 |  |  |
| Ig M | 0.63(0.42-0.95) | 0.027^*^ | 0.94(0.52-1.68) | 0.828 |
| Ig G | 1.07(1.00-1.14) | 0.068 | 0.98(0.86-1.11) | 0.696 |
| C3 | 1.00(1.00-1.00) | 0.126 |  |  |
| C4 | 1.00(1.00-1.00) | 0.288 |  |  |
| M0 | 1 (reference) |  |  |  |
| M1 | 1.07(0.39-2.97) | 0.896 |  |  |
| E0 | 1 (reference) |  | 1 (reference) |  |
| E1 | 1.91(1.02-3.57) | 0.044^*^ | 1.15(0.51-2.59) | 0.736 |
| S0 | 1 (reference) |  |  |  |
| S1 | 1.16(0.73-1.85) | 0.532 |  |  |
| T0 | 1 (reference) |  | 1 (reference) |  |
| T1-2 | 9.13(4.24-19.66) | <0.001^**^ | 2.79(1.08-7.25) | 0.035^*^ |
| C0 | 1 (reference) |  |  |  |
| C1-2 | 1.19(0.76-1.87) | 0.451 |  |  |

Abbreviations: MAP, mean arterial pressure; Scr, serum creatinine; eGFR, estimated glomerular filtration rate; BUN, blood urine nitrogen;UA,uric acid; Hb, hemoglobin; ALB, serum albumin; Glu, fasting glucose; CHOL, cholesterol; TG, triglyceride; LDL, low-density lipoprotein-cholesterol; HDL, high-density lipoprotein-cholesterol; SOD, superoxide dismutase; hsCRP, high sensitivity C reaction protein; ESR, erythrocyte sedimentatio rate; IgA, immunoglobulin A; IgM, immunoglobulin M; IgG, immunoglobulin G; C3, complement3; C4, complement4; M, mesangial hypercellularity; E, endocapillary hypercellularity; S, segmental glomerulosclerosis; T, tubular atrophy and interstitial fibrosis; C, crescents.

*P< 0.05, **P< 0.001.

**Supplement Table 4.** Clinicopathologic features as factors of elevated Hcy by univariate and multivariate logistic regression analysis in patients with eGFR≥90 ml/min/1.73 m^2^

Abbreviations: MAP, mean arterial pressure; Scr, serum creatinine; eGFR, estimated glomerular filtration rate; BUN, blood urine nitrogen;UA,uric acid; Hb, hemoglobin; ALB, serum albumin; Glu, fasting glucose; CHOL, cholesterol; TG, triglyceride; LDL, low-density lipoprotein-cholesterol; HDL, high-density lipoprotein-cholesterol; SOD, superoxide dismutase; hsCRP, high sensitivity C reaction protein; ESR, erythrocyte sedimentatio rate; IgA, immunoglobulin A; IgM, immunoglobulin M; IgG, immunoglobulin G; C3, complement3; C4, complement4; M, mesangial hypercellularity; E, endocapillary hypercellularity; S, segmental glomerulosclerosis; T, tubular atrophy and interstitial fibrosis; C, crescents.

| Variables | univariate |  | multivariate |  |
| --- | --- | --- | --- | --- |
|  | Odds ratio (95% CI) | *P* | Odds ratio (95% CI) | *P* |
| Age | 0.98(0.94-1.01) | 0.196 | 0.96(0.91-1.01) | 0.116 |
| Male | 3.62(1.80-7.31) | <0.001^**^ | 5.17(1.48-18.10) | 0.010^*^ |
| MAP | 1.02(0.99-1.05) | 0.118 |  |  |
| Scr | 1.09(1.06-1.13) | <0.001^**^ |  |  |
| eGFR | 0.94(0.91-0.97) | <0.001^**^ | 0.93(0.89-0.97) | 0.002^*^ |
| BUN | 1.44(1.08-1.93) | 0.014^*^ | 1.12(0.79-1.60) | 0.516 |
| UA | 1.00(1.00-1.01) | 0.131 |  |  |
| 24-h urine protein | 0.93(0.83-1.04) | 0.220 |  |  |
| Hb | 1.02(1.00-1.03) | 0.088 | 1.01(0.97-1.04) | 0.765 |
| ALB | 1.10(1.04-1.17) | 0.001^*^ | 1.10(1.03-1.18) | 0.005^*^ |
| Glu | 0.73(0.39-1.37) | 0.323 |  |  |
| CHOL | 0.87(0.72-1.04) | 0.122 |  |  |
| TG | 1.01(0.66-1.55) | 0.954 |  |  |
| LDL | 0.82(0.63-1.07) | 0.139 |  |  |
| HDL | 0.42(0.17-1.03) | 0.058 | 0.81(0.21-3.21) | 0.767 |
| SOD | 1.01(1.00-1.02) | 0.182 |  |  |
| hsCRP | 1.00(0.94-1.05) | 0.850 |  |  |
| ESR | 0.97(0.95-1.00) | 0.020^*^ | 1.01(0.97-1.04) | 0.690 |
| Ig A | 0.87(0.62-1.23) | 0.440 |  |  |
| Ig M | 0.74(0.43-1.27) | 0.274 |  |  |
| Ig G | 1.05(0.96-1.16) | 0.283 |  |  |
| C3 | 1.00(1.00-1.00) | 0.070 | 1.00(1.00-1.00) | 0.038 |
| C4 | 1.00(0.99-1.00) | 0.358 |  |  |
| M0 | 1 (reference) |  |  |  |
| M1 | 0.49(0.14-1.75) | 0.273 |  |  |
| E0 | 1 (reference) |  |  |  |
| E1 | 1.13(0.46-2.78) | 0.795 |  |  |
| S0 | 1 (reference) |  |  |  |
| S1 | 0.58(0.28-1.17) | 0.126 |  |  |
| T0 | 1 (reference) |  | 1 (reference) |  |
| T1-2 | 15.68(1.99-123.82) | 0.009^*^ | 11.36(1.32-97.88) | 0.027^*^ |
| C0 | 1 (reference) |  |  |  |
| C1-2 | 1.40(0.74-2.64) | 0.302 |  |  |

*P< 0.05, **P< 0.001.
